# Hospital Care for Jews in Nineteenth-century Amsterdam: The Emergence of the First Jewish Hospitals

**DOI:** 10.5041/RMMJ.10335

**Published:** 2018-04-19

**Authors:** Jack Y. Vanderhoek

**Affiliations:** Department of Biochemistry and Molecular Medicine, School of Medicine and Health Sciences, The George Washington University, Washington, DC, USA

**Keywords:** Amsterdam, Ashkenazi, hospital, Jewish, nineteenth century, Sephardi

## Abstract

In the early seventeenth century, the Jews formally established two separate communities in Amsterdam, the Portuguese Sephardi and the High German Ashkenazi congregations. Until the end of the eighteenth century, medical care for the Amsterdam indigent Jews had been controlled and regulated by the powerful *Parnasim*, the *de facto* rulers, of each community. The primary communal organizations that were exclusively responsible for medical care for the poor were the *Bikur Holim* societies. This approach for the care of the indigent Jewish sick became ineffective in the nineteenth century and was replaced by a hospital-based system. This essay describes how seriously ill indigent Jews in nineteenth-century Amsterdam received hospital care, tracing the establishment and development of the first Ashkenazi and Sephardi hospitals in the city. Although each community established their own hospital, they used different approaches to accomplish this goal.

## BACKGROUND

In the early seventeenth century, two groups of Jews received permission from the Amsterdam Magistrates to settle in their city. They were the Portuguese Sephardim, descendants of the Marranos, and the German Ashkenazim, refugees from various wars (e.g. the Thirty Years war) and pogroms.^1^ The Amsterdam city council granted both communities complete internal autonomy^2^,^3^ and held the governing *Parnasim* (community leaders;^4^ please see Box 1 for a Glossary) of each community *“*accountable to the city for the maintenance of law and order among the Jews.”^5^ (It should be noted that like Ashkenazi and Sephardi communities elsewhere in the world, the Amsterdam Ashkenazi and Portuguese Sephardi communities have maintained their separateness until today.) Practically, this meant that the *Parnasim* dominated every aspect of communal life as a result of their unlimited religious, juridical, and disciplinary powers. Furthermore, they also controlled nearly all welfare activities, including health, and ensured that taxes and other donations were collected to fund these activities.^3^^(p197),^^6^ Rather than trying to integrate a non-Christian group into their society, the city authorities opted to keep this group separate and avoid potential religious and social conflicts. As the Jews were primarily interested in preserving their religious and cultural traditions, they were willing to take on financial responsibility for all members, including their poor, in order to remain an autonomous community. This arrangement was fiscally advantageous to the city, and, with each side benefiting from this arrangement, its success was ensured.

Box 1 **Glossary**

(A): appended to a term: Ashkenazi community
(P): appended to a term: Portuguese Sephardi community
Ascamot: Laws and regulations
Bikur Holim: Visiting the sick; an important Jewish command (*mitzvah*)
Hekdesh: Community shelter and infirmary for the poor, transient, and sick
Parnasim: Community leaders
Meshiv Nefesh: Refreshing the soul
Mishenet Zekenim: Support for the aged
Mitzvah: Command
Nederlandsch Israelitisch Armbestuur (NIA): Dutch Israelite (Jewish) Poor Administration

The fact that Amsterdam was an important shipping port and international trading center made the city a very attractive destination for many Jews looking to improve their economic and religious situation.^6^^(p31)^ Both Portuguese Sephardi and Ashkenazi poor continued to immigrate and settle in Amsterdam during the seventeenth and eighteenth centuries, but the latter group increased at a much higher rate. From 1610 to 1650, the number of Portuguese Sephardim in Amsterdam increased from 350 to 1,400 and had doubled to 2,800 by 1750.^7^ During the same period, the Ashkenazi population increased from 0 in 1610 to 1,000 in 1650 and 14,000 in 1750.^7^

During the seventeenth and eighteenth centuries, the mechanism through which medical care of the sick, including the indigent, was provided was regulated by the *Parnasim* of each community. They used two approaches to fulfill this task. The first strategy involved the inclusion of “the appointments of physicians, surgeons, and apothecaries” as one of the articles in the Laws and Regulations of their synagogue,^8^ and the second approach was via the Laws and Regulations of the *Bikur Holim* society^4^^(s.v. “sick care, communal”)^ (a charitable society established in nearly all Jewish communities) of each community to ensure free medical care to their needy. For example, the 1703 *Ascamot* (Laws and Regulations) of the *Bikur Holim(P)* society delineated regulations and instructions for physicians hired by *Bikur Holim(P)*.^9^ The doctors were required to visit the ill poor at home, write prescriptions, and attend other patients (besides their own) in case of emergencies. Medical care for the Jewish poor was provided primarily by Jewish physicians and surgeons who visited and treated the ill in their homes.^10^,^11^ Home care was also the approach used to tend to the mentally ill.^6^^(p111)^ Sometimes the sick “were nursed … in private houses against payment by the Jewish community.”^12^

Although some of the early physicians provided their services gratis to the poor,^9^^(e.g. entry 334 no. 20, p. 109)^ eventually the *Parnasim* employed doctors full-time and paid their salary, thereby ensuring greater control.^6^^(p111),^^13^ Due to the sharp increase in the number of destitute Jews in the Ashkenazi community, the *Parnasim(A)* appointed three physicians in 1763 and five in 1773 to care for them.^10^

The major change in the nineteenth century that concerned the Dutch Jews was the direct involvement of the national government (in contrast to only municipal concerns in earlier times) with its reorganization of the Dutch Jewish community.^14^,^15^ In the century’s first decade, the government assumed responsibility for all matters outside the synagogue so that the *Parnasim*, including those of the Amsterdam communities, lost most of their power. As the *Parnasim* could no longer compel Jews to buy meat from the communal meat market (taxes generated from the sale of kosher meat constituted one of the major sources of communal income for the *Parnasim*),^6^^(p138)^ the financial condition of the Jewish community deteriorated and the whole organization of Jewish poor relief collapsed. In 1825, the government stepped in and established the Dutch Israelite (Jewish) Poor Administration (*Nederlandsch Israelitisch Armbestuur*, *NIA*) to manage all poor relief activities, including medical care, for the indigent Ashkenazi Jews.^12^^(p325),^^16^ Seven years later, the *NIA* became independent after it separated from the Amsterdam Ashkenazi community’s religious administration. The lay leadership of the *NIA* consisted of a board of directors who were prominent members of the Ashkenazi community and also included a physician. The institution was supported by funds from the main Ashkenazi synagogue, private donations, and subsidies from both municipal and national governments. The *NIA*’s primary focus was the Amsterdam Jewish Ashkenazi Hospital and Old Age Home.^16^^(pp58–9)^

As a result of the official separation of church and state in 1848, control over Dutch Jewish affairs was assumed by the Central Commission for Dutch Jewish Affairs (a non-governmental Jewish organization) which ultimately assumed supervisory control over the *NIA.*

Another major change was the economic situation of the Amsterdam Ashkenazi community. From 1809 to 1889, the city’s Jewish population (11%–13% of the total) increased from 21,444 to 54,479.^1^^(p90)^ During the first half of the century, almost two-thirds of Jews received poor relief.^14^^(p101)^ In 1809, 14% of the Amsterdam Ashkenazi Jews who received poor relief were ill or infirm.^17^ However, from 1866 to 1879, despite a very small increase in the number of Jews requiring poor relief (from 12,872 to 13,544), the proportion requiring poor relief due to sickness increased markedly (from 13% to 44%).^1^^(p92)^

Despite the government’s reorganization of the Dutch Jews into one community, the Portuguese Jews managed to maintain their separate status within this framework. In 1850, they reorganized their community’s governance so that their poor relief board was managed by four “daily” *Parnasim* together with a group of Regents from the Jewish community.^18^,^19^

Although the Jews were officially emancipated in 1796, the Amsterdam municipal authorities failed to eliminate many unfair and prejudicial regulations against the Jews.^14^^(pp54,57)^ For example, in an 1825 letter to the *Parnasim* of the Ashkenazi community, the mayor indicated that the city administration supported the decision of the Regents of the municipal hospitals and the city apothecary to exclude both Ashkenazi and Portuguese Jews from these institutions although no other residents were so disqualified.^9^^(entry 5186, no. 698, pp27–8,35–7)^ Claiming that prejudice was not involved, the mayor stated that this prohibition was based on the fact that the Jewish community was the only one to receive a municipal subsidy and that there already was a Jewish hospital. Although factually correct, it was well known that this hospital was unable to meet the demands of the Jewish community (see hospital discussion) and that the municipal subsidy was substantially less than that received by the Lutherans.^14^^(p97)^

In view of these noted changes in the nineteenth century, how did the Amsterdam Jewish community provide medical care for its seriously ill paupers?

## THE FIRST JEWISH HOSPITALS IN AMSTERDAM

**Figure 1:**
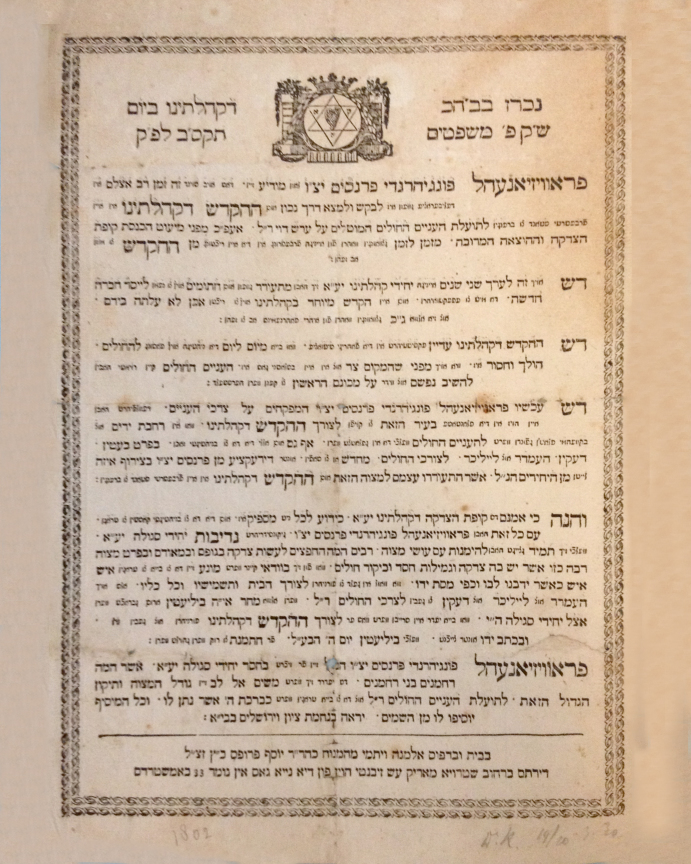
The Synagogue Announcement Requesting Financial Support to Build the First Jewish (Ashkenazi) Hospital in Amsterdam.^9^^(entry 714, no. 21)^ See Appendix.

The beginning of the nineteenth century was marked by both the precarious financial situation of the Jewish community and the increasing needs of the large number of Jewish indigent sick. As indigent Jews were not admitted to municipal hospitals (discussed above), six members of the Ashkenazi community (Messrs Alters, Cats, Dressau, Meijer,

Pinto, and Voet) decided that a Jewish hospital could provide better and more efficient care for the seriously ill destitute than the traditional one-on-one treatment of a patient by a district doctor who had to travel to the patient’s home.^20^ Furthermore, they realized that it would be much easier for lower-class Jews, who were generally ritually observant, to follow their religious practices and customs, including obtaining kosher food, among their co-religionists in a Jewish hospital than in a municipal facility. Finally, the success of the London Jewish Hospital (built in 1748) was also considered a favorable indicator. In 1802, an announcement (Figure 1)^9^^(entry 714, no. 21)^ was made in the Amsterdam main synagogue that the current *Hekdesh* (a communal shelter and infirmary for the poor, transient, and the sick)^4^^(s.v. “hekdesh”)^ was in poor condition and inadequate to meet the needs of the indigent sick. Consequently, the *Parnasim(A)* were requesting financial support to buy a spacious house and equipment to be converted into a hospital where sick paupers would be treated and recuperate. This appeal was successful.

In “early 1804 a hospital under Jewish management was established at Rapenburg,” even though it was considered rather “primitive.”^21^ (This land plot was located on the Rapenburgerstraat corner of Rapenburg and was part of the old Jewish neighborhood.) In the 1815 Regulations of the main Ashkenazi synagogue, the *Parnasim(A)* had maintained oversight over the Rapenburg hospital (Regulations, Article 37f).^22^ In fact, they had already realized that the Rapenburg building was quite dilapidated and were considering obtaining a new one (Article 186).^22^^(p56)^ In 1818 an unused old military hospital, situated on Roeters island, was purchased to replace the Rapenburg hospital and was inaugurated two years later.^20^^(pp20–1)^ However, even the Roeters island hospital was not quite suitable, and conditions became so bad that, in 1827, Dr D. Heilbron, the physician in charge, let it be known that “In a single ward patients, convalescents, lunatics and corpses are thrown together and the bunks are so close to one another that the doctors cannot move between them.”^12^

**Figure 2:**
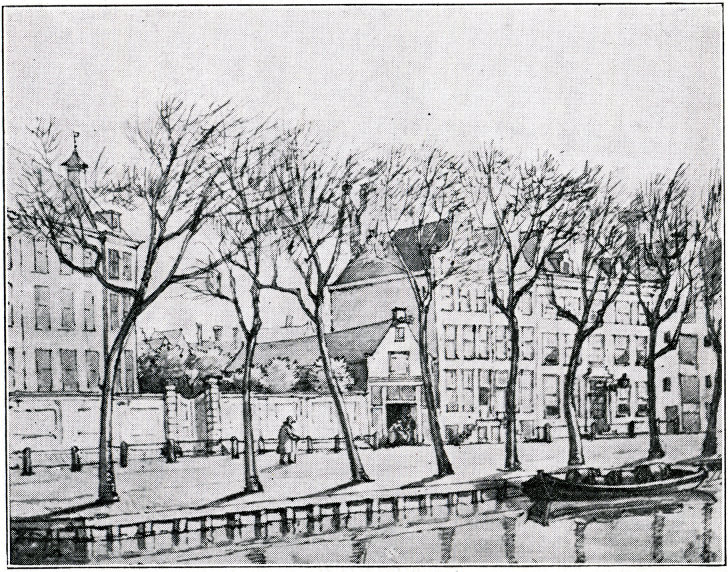
Picture of the First Amsterdam Jewish Ashkenazi Hospital and Old Age Home, Previously a Tavern Known as the Imperial Golf Course, Established in 1833.^16^^(p76)^

To improve this situation as well as to house the aged and infirm (such a combination was made mandatory by an 1825 Ministerial decree),^20^^(p23)^ a much larger building was required. In 1826, the *NIA* had taken over control of the Roeters island hospital from the *Parnasim(A)*^16^^(p101)^ and in 1832 had bought a parcel of land on the Nieuwe Kerkstraat (between the Weesperstraat and the Muidergracht) that housed a well-known tavern known as the Imperial Golf Course. Despite opposition from neighbors (who objected that the patients might disturb the “peace and quiet” of the neighborhood but who had not objected to the noisiness of the tavern),^20^^(pp25–6)^ the building was rebuilt and converted into the new Jewish Hospital and Old Age Home. The Jewish Ashkenazi Hospital and Old Age Home opened in 1833 (Figure 2)^16^^(p76)^ and continued to function until 1882.^21^

**Figure 3:**
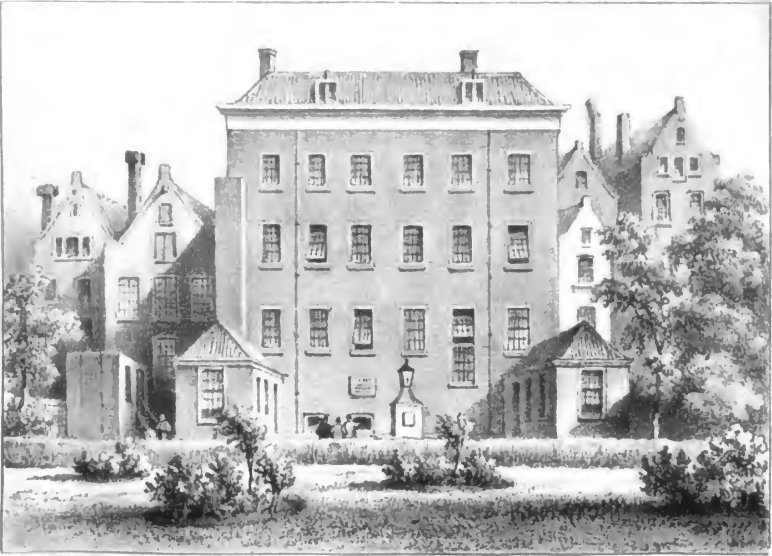
Picture of the 1840 Rebuilt Amsterdam Jewish Ashkenazi Hospital and Old Age Home as Seen from the Backyard.^23^

The top floors of the 1833 building were designated for use by the aged, and the lower floors housed the female patients; male patients and the mentally deranged were placed in an outbuilding in the backyard of the tavern. Sixty patients and 11 demented individuals could thus be treated. In 1834, an adjacent house was purchased which served to provide food for outpatients (provided by the *Bikur Holim(A)* society) and also to admit patients for whom there was no room in the main hospital. It soon became obvious that the whole facility was too small to meet the needs of the indigent Jewish sick. Two more adjoining houses were quickly procured which allowed a newly rebuilt and enlarged Jewish Hospital and Old Age Home (Figure 3)^23^ to be opened in 1840, located on the Nieuwe Kerkstraat, previously known as the Weesperveld.

The 1840 building consisted of four floors and contained 12 wards, seven for men and five for women, with a pharmacy on the ground floor.^18^^(pp221–2)^ Several rooms were used for operations and amputations, and there was separate accommodation for less needy sick patients as well as convalescents.^23^^(p67)^ The top floor housed the aged, and the one below was used for female patients.^24^ Needy patients with treatable illnesses were admitted, as were healthy paupers (if there was room), but patients who were also infected with scabies were not admitted and were nursed elsewhere.^18^^(pp221–2)^ Patients with eye diseases were kept in a separate ward. Physicians and surgeons were expected to file monthly reports describing the diseases that had been treated.

Another adjacent house was bought to contain the pharmacy on the ground floor; convalescent patients from the hospital resided here on the upper floors. By the early 1870s, the 1840 hospital had grown to house 98 patients, and it became obvious that the hospital was unable to treat more patients, especially because it lacked sufficient operating rooms and isolation wards.^16^^(pp101–4)^ Table 1 describes some typical patient numbers in the Dutch Jewish Ashkenazi Hospital and Old Age Home during the nineteenth century. Except for the 1875 statistics, the number of patients treated in this hospital kept increasing from 1845 through 1880.

**Table 1:** Comparative Patient Numbers in the Dutch Jewish Ashkenazi Hospital and Mental Hospital during the Nineteenth Century.

Institution	1845	1850	1860	1875	1880
**Hospital**	Ref. 25	Ref. 18(p223)	Ref. 26	Ref. 27	Ref. 27
In-patients	69	71	94	69	61
Newly admitted	344	377	398	302	581
Discharged	273	310	344	242	521
Died	69	58	72	64	54
**Mental hospital**	Ref. 25	Ref. 18(p241)	Ref. 26(p32)	Ref. 27(p39)	Ref. 27(p39)
In-patients	13	29	63	134	144
Newly admitted	6	16	16	36	41
Discharged/cured	3	7	12	21	29
Died	6	6	10	14	17

During the last quarter of the nineteenth century, there was a tremendous increase in the number of sick paupers requiring medical assistance. Hence, it was decided in 1878 to convert the hospital building exclusively into an Old Age Home and to build a state-of-the-art hospital on the site where the original Old Age Home and three warehouses had been located. After much planning, a newly rebuilt and expanded Ashkenazi Jewish hospital was opened in 1882 which housed 110 patients and incorporated the modern developments of the late nineteenth century in hospital practice. These included a surgical department with separate septic, aseptic, and sterilization rooms, isolation wards for contagious diseases (e.g. rooms for tuberculosis patients had large balconies to facilitate “open air” treatments), separate wards for pregnant and nursing women, a nurses’ home to train nurses (a relatively new field of medical practice), a separate X-ray department (started in the early twentieth century), outpatient clinics (to treat surgical problems, eye and ENT diseases, pediatric ailments, obstetrics and emergency conditions), and a pharmacy.^21^ Two years later this hospital was considered the “best” hospital in Amsterdam.^16^^(p107)^

Another group that needed to be cared for was the indigent mentally ill. Until the nineteenth century, treatment of patients with mental disorders was inhuman.^28^ As discussed above, the 1833 Jewish Ashkenazi hospital used part of an outbuilding in the backyard of the original tavern for male patients, and the other part was adapted to house both male and female mental cases, although very few mentally ill patients could be accommodated.

The passage of the 1841 law regulating care for demented individuals, such as separation of men and women and the availability of daily activities and diversions, spurred the *NIA* to purchase additional buildings to expand the hospital facilities. One of these was a house on the Keizersgracht near the Weesperstraat, named the Shield of Turnhout, which was renovated, inaugurated in 1856, and used to care for female mental patients; the outbuilding that was part of the Imperial Golf building was converted to treat mentally disturbed men.^28^ The patients were treated according to their conditions and the latest medical knowledge. During this time, the typical treatment changed from a “restrained” approach (i.e. patients were chained and locked in small cubicles) to the “not-restrained” system. Due to the increase in size, other Jewish communities were urged to send their mentally handicapped patients to be admitted to this facility so that the patients would remain in a Jewish environment.^16^^(p128)^

Table 1 shows typical patient numbers during this time. From 1845 to 1880, there was a steady increase in the number of mental patients treated at this facility. In 1855, the Shield of Turnhout building was changed to accommodate male mental patients. After the purchase of additional buildings, a new and enlarged women’s ward was inaugurated in 1865. At this time about 90 patients were treated, and by 1888 this number had increased to 144. The rather large increase in patient numbers in the 1870s and 1880s was probably due to the fact that the hospital was open not only to Amsterdam patients but also to Jewish patients from other Dutch cities whose families wanted a Jewish environment where religious laws and customs were followed. In 1882, 60% of the patients were from Amsterdam.^27^^(pp37–8)^ Toward the end of the century, medical ideas in treatment changed again, the most important being bed rest.^29^ Other changes included the ability of patients in the Jewish Mental Hospital (Asylum) ward to engage in handcrafts, games, and gymnastics and to use a well-stocked library. Several experts from Belgium and Germany reported that this mental hospital was an outstanding institution.^26^^(p31)^ The Dutch Jewish Ashkenazi Asylum continued to function until 1921 when the patients were all transferred to the Jewish Psychiatric institute “Het Apeldoornsche Bosch” in Apeldoorn.

As for the Dutch Portuguese Jewish community, we have noted above that a majority of this group was also dependent on poor relief, although their numbers were much smaller than those for the Ashkenazi indigent. However, it would take about 30 years after the start of the first Ashkenazi hospital before the Portuguese Jewish community decided to establish their own institution to provide medical care for their indigent. Several reasons could explain this delay. Perhaps the achievements of both the Ashkenazi Amsterdam hospital as well as the London Jewish hospital of the Spanish-Portuguese community led some progressive community members as well as the Jewish physicians treating the ill Portuguese indigent to finally conclude that these patients would receive better treatment in a hospital setting. Another consideration was that there was an increased need to care for healthy but poor old women (an old-age institution for men—*Mishenet Zekenim* [Support for the aged]—had already been established in 1749),^19^^(p161)^ and obtaining a building for them which would include a hospital wing for the indigent would simultaneously solve both problems.

**Figure 4:**
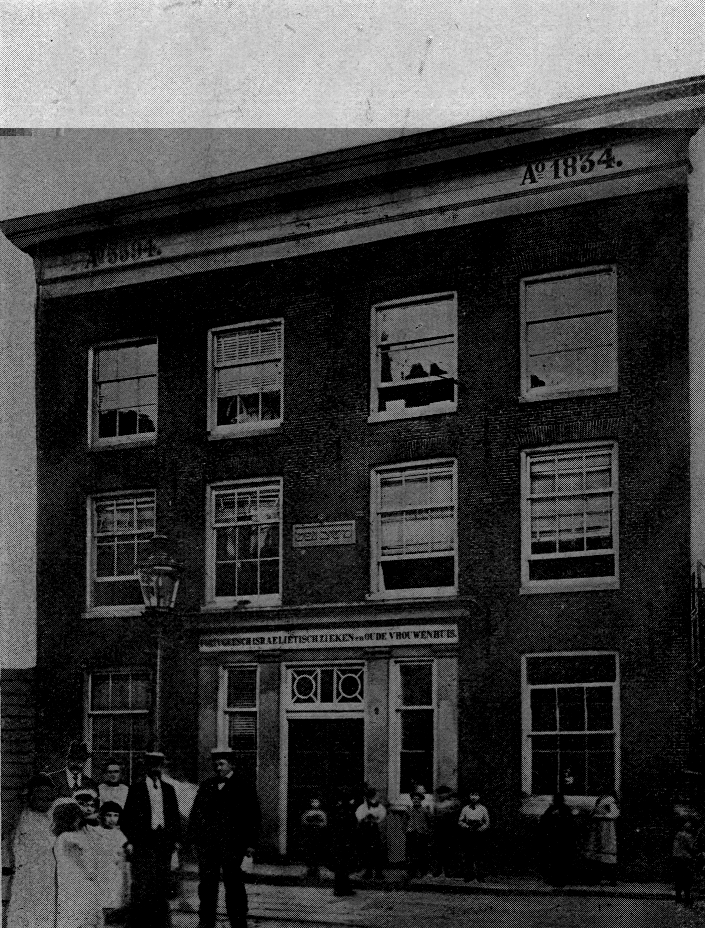
Picture of the First Portuguese Jewish Hospital and Old Age Home for Women Which Was Established in 1834.^19^^(p23)^

However, the Portuguese Jews decided to use a different approach (from their Ashkenazi neighbors) to support these two groups. First of all, they did not establish a separate poor relief organization such as the *NIA*. Second, I have not uncovered any record in the archives that a public appeal for funds was made to start and maintain a hospital. It is likely that enough monies (and/or donors) were available since the archival records contain a list of 269 contributors (pledging *fl.* 9611 in outright donations and *fl*. 1332 in annual commitments) to the establishment of a hospital.^9^^(entry 334, no. 1276)^ Third, they ensured that the religious lay leadership (i.e. the *Parnasim(P)*) maintained control of this new charitable organization. In 1833, they established the *Meshiv Nefesh* (Refreshing the Soul) institution where both ill paupers could be tended and healthy old women could spend their last years care-free. A land parcel at the Rapenburgerstraat corner of Rapenburg, that was the original site of the first Ashkenazi Jewish hospital (see above), was purchased and the building was inaugurated in 1834.^19^^(p161)^ Figure 4 shows a picture of this hospital during its existence from 1834 to 1916.^19^^(p23)^

According to the 1834 Regulations of *Meshiv Nefesh*,^9^^(entry 334, no. 1277, pp1–4)^ the function of this hospital was to treat the indigent in- and outpatients and maternity cases as well as to care for the healthy female elderly of the Portuguese Jewish community (Art. 1). Its board of five directors was chosen at a general meeting of both the governing board of the *Bikur Holim(P)* and the *Parnasim(P)* (Art. 5). All had to be members of the Portuguese Jewish community (Art. 1) and were chosen for a five-year term from the community’s elders and could be re-elected (Art. 2, 4).

The appointments of the physicians, surgeons, and apothecaries (with preference given to community members who had the necessary qualifications) were made at a meeting of the *Meshiv Nefesh* governing board in consultation with the *Parnasim(P)* (Art. 14, 15). The directors also had to make sure that only indigent patients who belonged to the Portuguese community could be treated (Art. 18). In 1842, the Regulations were updated, although the main guidelines remained the same. For example, only those patients could be admitted whose recovery would be likely. Patients with contagious diseases (e.g. measles, small-pox, or scarlet fever) were excluded (Art. 32).^9^^(entry 334, no. 1277, pp26–7)^ However, the indigent, including maternity cases, who could not be admitted to the institution, would get free medical homecare and medicines (Art. 35).^9^^(entry 334, no. 1277, pp26–7)^ Specific instructions for physicians, surgeons, and apothecaries were also described.^9^^(entry 334, no. 1277, pp36–41,50–2)^ These included visiting hours for in- and outpatients, vaccination of children, consultations with academics regarding unusual cases, and the maintenance of proper records.

As a result of the disbanding of the *Bikur Holim(P)* in 1848, and the passage of the Separation of Church and State law in the same year, control and financial support of *Meshiv Nefesh* came under the sole purview of the *Parnasim(P).* Several *Parnasim(P)* and the board of Governors were responsible for the daily management of the institution. In 1850, this institution treated on average six patients and cared for 12 old women.^18^^(pp210,224)^ This hospital remained in use until 1916 when a new Portuguese Jewish hospital (on the Plantage Franschelaan) was dedicated.

Throughout most of its history, one of the striking features of the Amsterdam Jewish community was the poverty of most of its members. Although various Jewish charitable societies were instrumental in providing some sort of poor relief, the Jewish indigent sick were the most vulnerable segment of the community. In order to meet the needs of this group, the *Bikur Holim* societies of the seventeenth and eighteen centuries of the Ashkenazi and Portuguese Jewish communities were established exclusively for providing medical care for the sick Jewish indigent paupers. In the nineteenth century, the Jewish hospital fulfilled this role of being “entirely at the disposal of the poor and indigent.”^21^ Other Dutch Jewish communities (except in The Hague) had neither the population numbers nor the financial resources to establish a Jewish hospital but relied on their *Bikur Holim* societies to provide medical care and assistance for their destitute sick.

The question arises whether other religious communities in Amsterdam followed the example of their Jewish neighbors and established hospitals for the poor. Since assisting the destitute was also a religious responsibility for all Christian denominations, they organized and administered their own poor relief organizations for many centuries.^30^ These organizations provided free (or low-cost) medical care for their sick and needy members. Such medical care consisted primarily of home visits by physicians or surgeons as well as free medicines^31^ and was similar to the services provided by the *Bikur Holim* societies. In contrast to the founding of the Jewish hospitals in the early nineteenth century, the establishment of the Christian denominationally supported hospitals did not occur until the end of the nineteenth century and did not seem to be solely centered on medical care for the indigent. Treatment in one of the two municipal hospitals—the Binnengasthuis (Inner hospital) and the Buitengasthuis (Outer hospital)—was initially restricted to those Protestant^31^ poor without any religious affiliation who could not be nursed at home or had to be isolated because of a contagious disease. Perhaps the various Christian denominations were satisfied that their medical poor relief organizations were able to adequately meet the needs of their sick indigent co-religionists. Near the end of the century, there also was an attitudinal shift towards the hospital. Whereas hospitals (both Jewish and municipal) were originally designed for charity cases, the lower middle class started to accept the idea that hospitals could provide better treatment for certain illnesses and medical conditions than home care. Several reasons can explain this shift, including improved medical care (both preventive and curative) and decreased death rates in hospitals, stricter standards for physicians and surgeons, more governmental oversight and regulations (e.g. better hygiene), and a rising standard of living which enabled people to obtain health insurance for hospitalization. These changes made the hospitals no longer the sole purview for the indigent. By the end of the nineteenth century, several denominational hospitals were established, including the Catholic Holy Virgin hospital, the Dutch Reformed Deaconess Institution, and the Lutheran Deaconess Institution.^21^^(pp47,87,93)^

The primary stimuli for the establishment of the first Ashkenazi and Sephardi hospitals in nineteenth-century Amsterdam were the large number of seriously ill indigent Jews as well as the lack of financial resources in the Jewish community. Differences in patient numbers, communal and governmental resources, and hospital leadership determined the path each community took to establish and develop their own hospitals and meet the medical needs of their indigent sick co-religionists.

## Supplementary Information



## Abbreviations

NIA: Nederlandsch Israelitisch Armbestuur.
