# Supplementary material for: Hospital Care for Jews in Nineteenth-century Amsterdam: The Emergence of the First Jewish Hospitals
Source: Rambam Maimonides Med J. 2018 Apr 19;9(2):e0016. doi: 10.5041/RMMJ.10335 (PMC5916234; doi:10.5041/RMMJ.10335)
Supplement: Supplementary file 1 [file rmmj-9-2-e0016-sup.pdf]

SUPPLEMENTARY MATERIAL

This appendix has been provided by the authors to give readers additional background reading and information

Supplement to Hospital Care for Jews in Nineteenth-century Amsterdam: The Emergence of the First Jewish Hospitals

Vanderhoek JY. Hospital Care for Jews in Nineteenth-century Amsterdam: The Emergence of the First Jewish Hospitals. Rambam Maimonides Med J 2018;9 (2):e0016. doi:10.5041/RMMJ.10335

APPENDIX

TRANSLATION OF THE APPEAL MADE IN THE ASHKENAZI SYNAGOGUE IN AMSTERDAM IN 1802 TO ESTABLISH A HOSPITAL FOR ASHKENAZI SICK PAUPERS

"Proclaimed in the synagogue of our congregation on the holy Shabbat Parshas Mishpatim 5562 (30 Jan 1802).

"The Provisionally acting *Parnasim* (may their Rock and Redeemer protect them) announce that although for a long time they have deliberated to try to find the right way to improve the *Hekdesh* of our congregation for the benefit of the sick paupers who are cloistered to their deathbed (may Heaven save them).

"Nevertheless, because of the meager income to the Poor fund and its many expenditures, they were occasionally forced to refrain from any improvement in the facilities of the *Hekdesh*.

"Also some noble *Kehilla* [congregation] members (may the Supreme One favor them) have come forward and tried for about two years to collect signatories in order to establish a new [charitable] society to bring about the establishment of a special *Hekdesh* in our congregation. However, they were not successful and they were forced to abandon their plans.

"The *Hekdesh* of our congregation still exists in its previous condition whereby the needed supervision of the sick decreases daily. Since the place is limited and is [located] on a closed street, the destitute sick have no room to recuperate (to refresh their Souls), and to be restored to their former state.

"That now the Provisionally acting *Parnasim* (may their Rock and Redeemer protect them) who have the oversight for providing the necessary supplies for the poor, have decided to buy a house in the Plantage [part] of this city [to function] as a *Hekdesh* of our congregation that is spacious and in good condition to

which the sick paupers can be admitted. To also purchase all necessities for the sick such as beds, blankets, shirts and pajamas [?] [which will be carried out] under the direction of the *Parnasim* in collaboration with several of the aforementioned noble members who had come forward to fulfill this *mitzvah*, i.e. to improve the *Hekdesh* of our congregation.

“And now, as the Poor fund of our *Kehilla*—as everyone knows—is not sufficient to carry the necessary costs, the Provisionally acting *Parnasim* have hereby considered the contributions of the noble people who have always demonstrated to be counted among the *Mitzvah*-doers. There are many who want to do kindness with both their person and their assets and especially for such a great *mitzvah* that combines kindness, charity and visiting the sick.

“There is certainly no one who does not want to contribute to this: everyone as his heart urges him and as his capabilities permit him, both in donating money needed to purchase the house, its equipment and all its consumables, as well as shirts, and pajamas [?] and blankets needed for the sick.

“Tomorrow (with God’s help) the noble members will distribute forms on which everyone can describe what he can furnish and provide for the infirmary of our congregation, to be signed by his own hand. These forms will be collected next Thursday.

“The Provisionally acting *Parnasim* are assured of the benevolence of the noble members who are merciful and are the sons of merciful ones, and of every person who takes to heart the magnitude of this *mitzvah* and this great sublimation for the benefit of the destitute sick and so will contribute to the blessing that the Eternal One has given him and to all who still will contribute will also be awarded by Heaven and he will experience the consolation of Zion and Jerusalem speedily, even in our days.

“(Printed in the printing house of the widow and orphans of Yosef Proops Katz. Their home is located on Stroo market in the 7th house of the new street at #33, Amsterdam.)”
